# A signature of immune-related genes correlating with clinical prognosis and immune microenvironment in sepsis

**DOI:** 10.1186/s12859-023-05134-1

**Published:** 2023-01-17

**Authors:** Zhong-Hua Chen, Wen-Yuan Zhang, Hui Ye, Yu-Qian Guo, Kai Zhang, Xiang-Ming Fang

**Affiliations:** 1 Department of Anesthesiology and Intensive Care, The First Affiliated Hospital, School of Medicine, Zhejiang University, QingChun Road 79, Hangzhou, 310003 China; 2 Department of Anesthesiology, Shaoxing People’s Hospital, Shaoxing, China

**Keywords:** Clinical prognosis, Immune microenvironment, Immune-related genes, Sepsis, Signature

## Abstract

**Background:**

Immune-related genes (IRGs) remain poorly understood in their function in the onset and progression of sepsis.

**Methods:**

GSE65682 was obtained from the Gene Expression Omnibus database. The IRGs associated with survival were screened for subsequent modeling using univariate Cox regression analysis and least absolute shrinkage and selection operator in the training cohort. Then, we assessed the reliability of the 7 IRGs signature's independent predictive value in the training and validation cohorts following the creation of a signature applying multivariable Cox regression analysis. After that, we utilized the E-MTAB-4451 external dataset in order to do an independent validation of the prognostic signature. Finally, the CIBERSORT algorithm and single-sample gene set enrichment analysis was utilized to investigate and characterize the properties of the immune microenvironment.

**Results:**

Based on 7 IRGs signature, patients could be separated into low-risk and high-risk groups. Patients in the low-risk group had a remarkably increased 28-day survival compared to those in the high-risk group (*P* < 0.001). In multivariable Cox regression analyses, the risk score calculated by this signature was an independent predictor of 28-day survival (*P* < 0.001). The signature's predictive ability was confirmed by receiver operating characteristic curve analysis with the area under the curve reaching 0.876 (95% confidence interval 0.793–0.946). Moreover, both the validation set and the external dataset demonstrated that the signature had strong clinical prediction performance. In addition, patients in the high-risk group were characterized by a decreased neutrophil count and by reduced inflammation-promoting function.

**Conclusion:**

We developed a 7 IRGs signature as a novel prognostic marker for predicting sepsis patients’ 28-day survival, indicating possibilities for individualized reasonable resource distribution of intensive care unit.

**Supplementary Information:**

The online version contains supplementary material available at 10.1186/s12859-023-05134-1.

## Introduction

Sepsis is a major public health problem on a global scale and one of the main causes of death in intensive care units (ICU) [1, 2]. At least 5.3 million patients in the world are estimated to be diagnosed with sepsis each year, and the mortality of these patients remains approximately 30% [3–5]. The high mortality of sepsis patients is largely attributed to lack of accurate methods for early prediction of clinical outcome [6]. Increasing evidence [7, 8] indicates that the systemic immune response has a critical function to play in the pathogenesis and progression of sepsis. In the initial phase of sepsis, the immune response is dominated by pro-inflammatory processes and is favorable for the eradication of pathogens [9]. Progressive sepsis is mainly characterized by the suppression of the immune response, as seen by a decline in the function and number of immune cells [10]. The poor prognosis of sepsis may also be closely related to a compromised host immune system [11, 12], and more and more studies [13, 14] have suggested that novel immune biomarkers cannot only serve as potential predictors of sepsis prognosis but also can provide potential targets for immunotherapy of sepsis. Thus, it becomes necessary to explore immune biomarkers deeply to improve the clinical management of sepsis patients and their prognosis.

The transcriptomic research landscape has undergone a paradigm shift as a result of recent developments in high-throughput, next-generation sequencing and gene chips technology [15]. Numerous bioinformatics analyses and machine learning analyses have been conducted to explore the mRNA prognostic signatures and to direct clinical practice [16, 17]. Prognostic signatures based on immune-related genes (IRGs) have been described for a variety of types of cancer and have demonstrated high sensitivity and specificity [18–20]; however, these signatures have not been applied to predict the outcome of patients with sepsis. As a result, we sought to develop and validate an IRGs signature for predicting sepsis patient prognosis and to characterize the immune microenvironment in sepsis patients with varying prognostic risk.

In the present investigation, we conducted a systematic analysis of the immunogenomic landscape of sepsis using Gene Expression Omnibus (GEO) gene expression profiles, and we identified 7 IRGs. In both the training and validation cohorts, a unique IRGs prognostic signature was established and showed moderate predictive value for sepsis patient survival. Furthermore, the CIBERSORT algorithms and single-sample gene set enrichment analysis (ssGSEA) results showed that the high-risk group of sepsis patients were characterized by a decreased neutrophil count and by reduced inflammation-promoting function.

## Methods

### Acquisition of gene expression profiles and clinical information

**Fig. 1:**
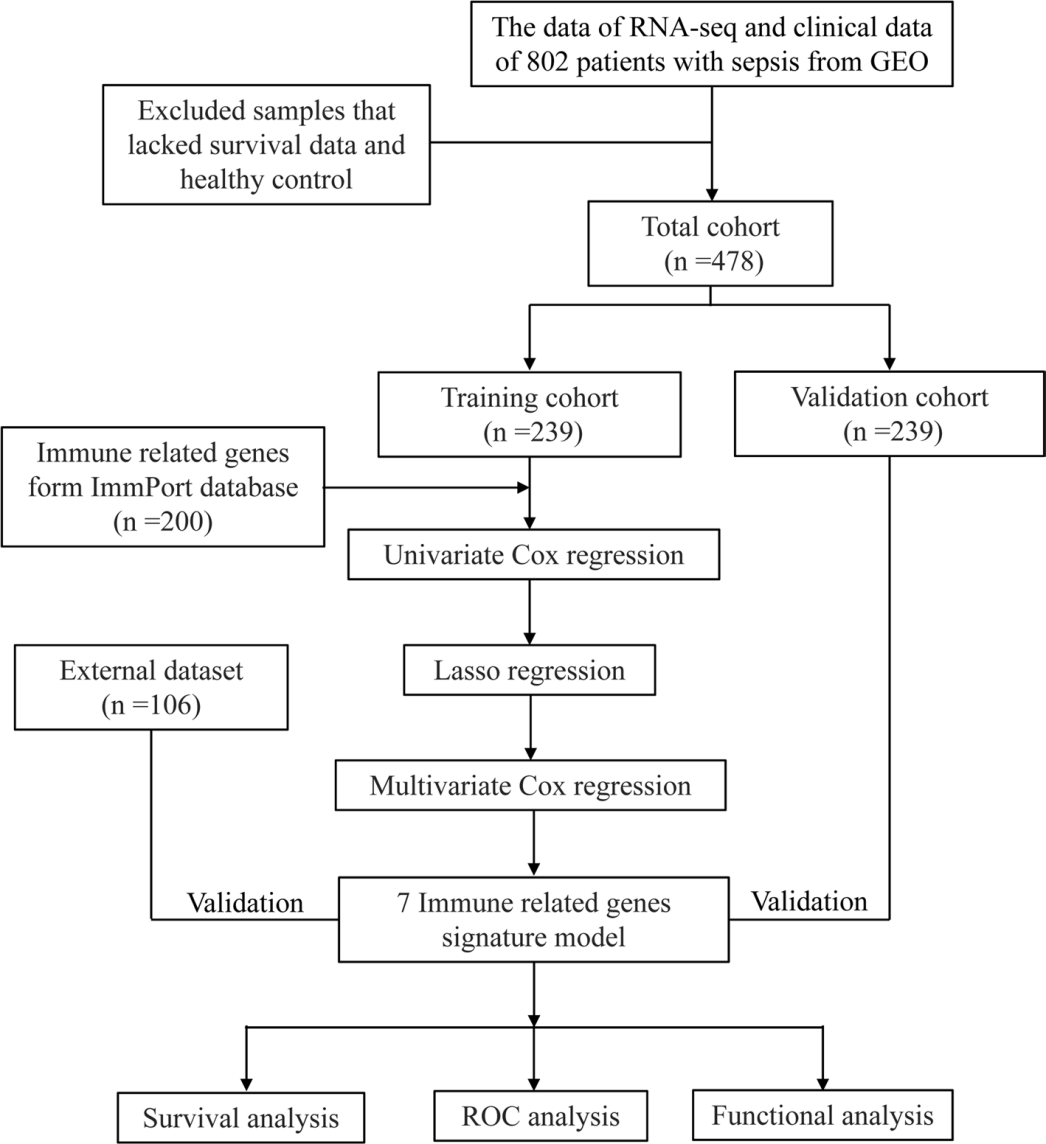
The study's flow chart. *LASSO* least absolute shrinkage and selection operator, *ROC* receiver operating characteristic curve, *GO* gene ontology, *KEGG* Kyoto encyclopedia of genes and genomes

GEO database (https://www.ncbi.nlm.nih.gov/geo/) [21] was the source of the level 3 RNA sequencing (RNA-seq) data and related clinical information of 802 sepsis patients (GSE65682 [22]). The clinical information encompassed age, gender, pneumonia, thrombocytopenia, ICU acquired infection, diabetes, abdominal sepsis, survival status and survival time. The healthy controls and sepsis patients with unavailable survival data were excluded. In the end 478 sepsis patients with integrated RNA-seq data and clinical information were screened. In the ArrayExpress database [23], the high-throughput sequencing data of E-MTAB-4451 dataset [24] and prognostic information were selected as external data sets to verify and analyze the model. The E-MTAB-4451 dataset contained a total of 114 samples of adult patients with sepsis. After excluding sepsis samples with incomplete clinical data, 106 sepsis samples were finally included. All the above samples were obtained from peripheral blood samples of septic patients within 24 h after admission to ICU. Subsequently, we performed principal components analysis (PCA) analysis on the expression values of the samples after batch correction. The workflow sketched in Fig. 1.

### Identification of the immune-related genes

The Immunology Database and Analysis Portal (ImmPort) database (https://www.immport.org/) was employed to located IRGs. The ImmPort database had 2498 IRGs, which were listed in Additional file 1: Table S1.

### Construction and validation of a prognostic immune-related genes signature

Four hundred and seventy-eight sepsis patients were randomly separated into two groups, one for training and one for validation, in a ratio of 1:1. Firstly, to assess the connections between IRGs and patient survival outcomes in the training cohort, univariate Cox regressions were performed. Secondly, the prognostic IRGs were recognized utilizing least absolute shrinkage and selection operator (LASSO) Cox regression [25], which reduced the number of IRGs with prognostic values. In addition, an interaction network of IGRs was created by the STRING database [26]. Thirdly, multivariable Cox regression analysis was employed to identify prognostic IRGs. Seven significant IRGs associated with survival were discovered according to the least Akaike information criterion (AIC) value. AIC was mainly used to measure the goodness of fit of the statistical model, and the model with the lowest AIC was the prediction model with the best fit. Fourth, according to the risk score, sepsis patients in the training and validation cohorts were separated into low-risk and high-risk groups. The following formula was used to determine the risk score: risk score = *β*gene (a) × *EXP*gene (a) + *β*gene (b) × *EXP*gene(b) + …  + *β*gene(n) × *EXP*gene(n), with *EXP*gene representing the expression level of the identified IRGs standardized by Z score and *β* representing the coefficient of that particular IRGs in multivariable Cox regression analysis. Kaplan–Meier analysis was performed with the R package “survival”, as well as “survminer”, to compare survival between low-risk and high-risk groups. For the purpose of assessing the accuracy of the constructed signature, the “pROC” R package was applied. Univariate and multivariable Cox regression analyses were recruited to evaluate the signature's capacity to serve as an independent prognostic yardstick in comparison to clinical features in the study population.

### Enrichment analyses of the differentially expressed genes (DEGs)

The DEGs between low-risk and high-risk groups were obtained using the “limma” R package [27]. |log2FC|≥ 1 and *P* < 0.05 were regarded as the cutoff criterion for DEGs. Gene Ontology (GO) and Kyoto Encyclopedia of Genes and Genomes (KEGG) analysis was performed using the "clusterProfiler" R package based on DEGs [28]. The gene set enrichment analysis (GSEA) (http://software.broadinstitute.org/gsea/index.js) was used to compare inflammatory pattern in the different risk groups.

### Comprehensive analysis of immune status

The CIBERSORT algorithms [29] were employed to compare the fraction of immune cells between low-risk and high-risk groups. Furthermore, with ssGSEA, we estimated the infiltration score for the activity of 13 immune-related pathways [30]. Prospective immunological check-point genes were described in research articles.

### Statistical analysis

Chi-squared tests were conducted to compare differences in proportions. Univariate and multivariable Cox regression analyses were implemented to distinguish IRGs linked with prognosis. The Kaplan–Meier analysis and the log-rank test were performed to compare the survival between low-risk and high-risk groups. The ssGSEA scores of immune cells or pathways were compared between the low-risk and high-risk groups using Mann–Whitney *U* test with *P* values adjusted by the Bonferroni-Holm (BH) method. The prognostic prediction signature's predictive accuracy was measured via receiver operating characteristic curve (ROC) analysis. All of these analyses entailed the use of SPSS software 23.0 and R software 4.0. *P* value less than 0.05 was considered to be statistically significant.

## Results

### The clinical information of the training and the validation cohort

Age, gender, the type of pneumonia, the proportion of thrombocytopenia, the proportion of ICU-acquired infection, the proportion of diabetes, the proportion of abdominal sepsis were not significantly different between the training and the validation cohort (*P* ≥ 0.05) (Table 1).

**Table 1:** The clinical characteristics of sepsis patients in the training and the validation cohort

Variables	Training cohort (*n* = 239)		Validation cohort (*n* = 239)		*P*
	No	%	No	%	
*Age (y)*					
≤ 65	138	57.74	134	56.07	0.782
> 65	101	42.26	105	43.93	
*Gender*					
Female	100	41.84	106	44.35	0.644
Male	139	58.16	133	55.65	
*Pneumonia*					
Community acquired pneumonia	58	24.27	48	20.08	0.442
Hospital acquired pneumonia	40	16.74	37	15.48	
Unknown	141	58.99	154	64.44	
*Thrombocytopenia*					
Yes	45	18.83	37	15.48	0.396
No	194	81.17	202	84.52	
*ICU-acquired infection*					
Yes	23	9.62	23	9.62	1.000
No	216	90.38	216	90.38	
Diabetes					
Yes	42	17.57	47	19.67	0.639
No	197	82.43	192	80.33	
*Abdominal sepsis*					
Yes	27	11.30	22	9.21	0.547
No	212	88.70	217	90.79	

*ICU* Intensive care unit

### Construction of a prognostic immune-related genes signature

**Fig. 2:**
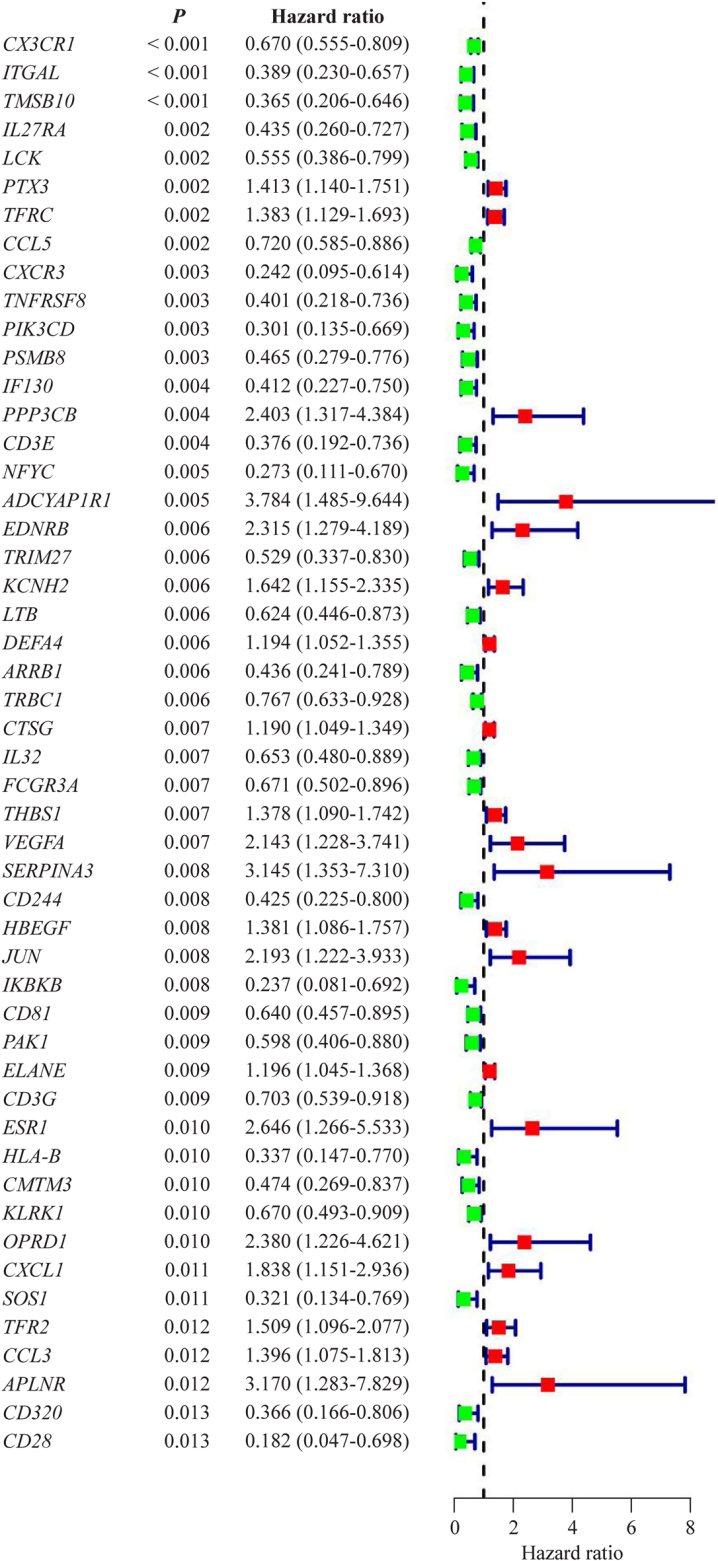
Identification of the candidate top 50 immune-related genes associated with survival of sepsis patients

**Fig. 3:**
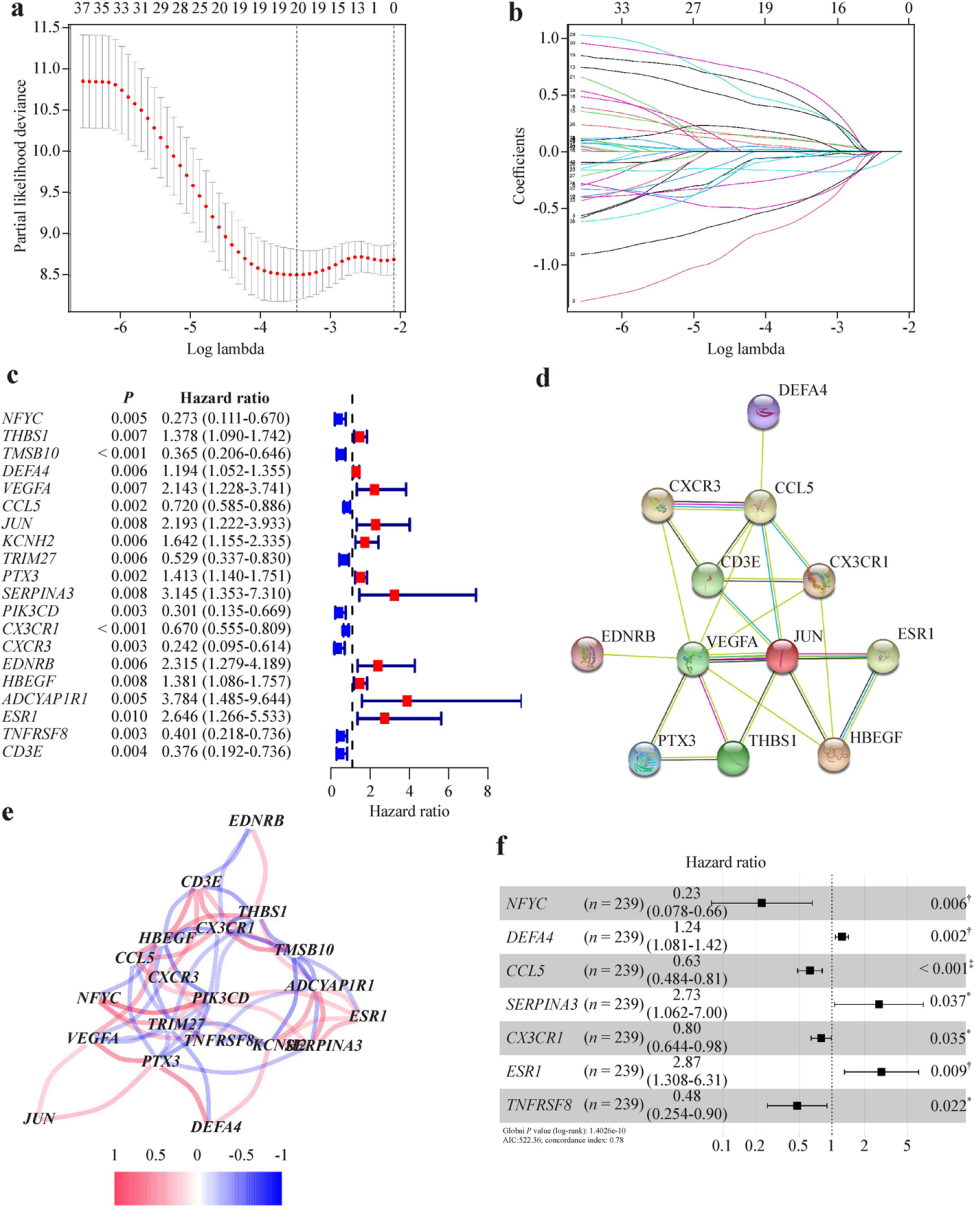
An immune-related genes (IRGs) prognostic signature was constructed in sepsis patients. **a** 20 IRGs were screened by the LASSO regression model; **b** LASSO Cox regression analysis was used to compute the coefficients of IRGs; **c** forest plots illustrating the findings of a univariate Cox regression analysis examining the relationship between the 20 IRGs and 28-day survival in patients with sepsis; **d** the interactions of candidate IRGs are depicted in the protein–protein interaction network; **e** the correlation network of candidate IRGs; **f** forest plots illustrating the findings of a multivariable Cox regression analysis examining the relationship between the 20 IRGs and 28-day survival in patients with sepsis. *LASSO* Least absolute shrinkage and selection operator, *AIC* Akaike information criterion. ^*^P < 0.05, ^†^*P* < 0.01, ^‡^*P* < 0.001

**Fig. 4:**
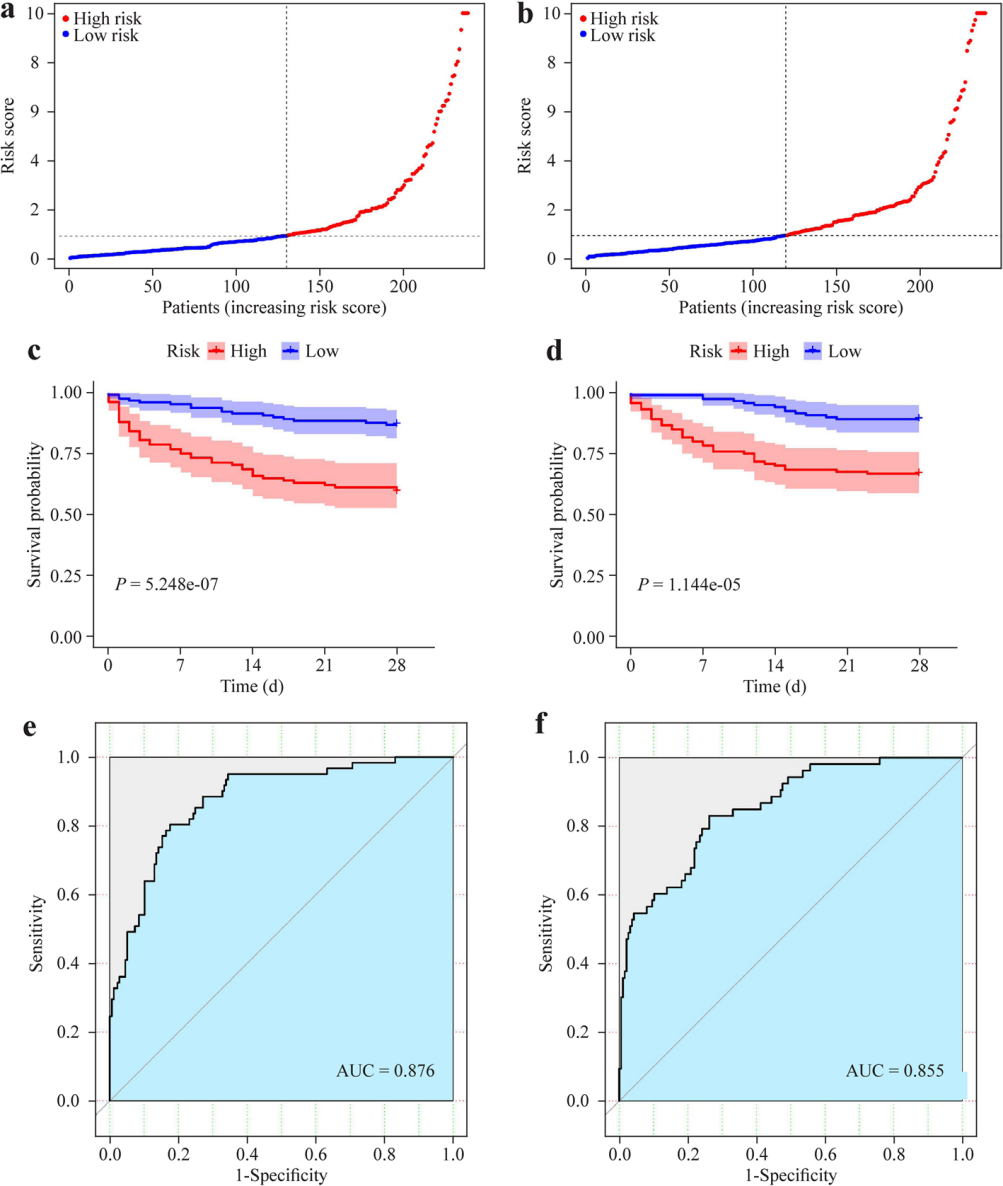
The 7 immune-related genes (IRGs) signature's prognostic performance in the training cohort and the validation cohort. **a** The median value and distribution of the risk scores calculated by this signature in the training cohort; **b** the median value and distribution of the risk scores calculated by this signature in the validation cohort; **c** Kaplan–Meier curves depicting the 28 days survival based on the risk score in sepsis patients in the training cohort; d Kaplan–Meier curves depicting the 28 days survival based on the risk score in sepsis patients in the Validation cohort; e the prognostic accuracy of the risk score is confirmed by the AUC of ROC curves Kaplan–Meier curves depicting the 28 days survival based on the risk score in sepsis patients in the training cohort; **f** the prognostic accuracy of the risk score is confirmed by the AUC of ROC curves Kaplan–Meier curves depicting the 28 days survival based on the risk score in sepsis patients in the validation cohort. *AUC* Area under curve, *ROC* Receiver operating characteristic curve

We conducted PCA analysis on the expression values of the samples after batch correction (Additional file 2: Fig. S1a, b). A total of 752 IGRs were expressed in sepsis patients. Firstly, following the univariate Cox regression analysis, we identified 126 IRGs that were linked with survival and had prognostic significance (Fig. 2, Additional file 1: Table 1, *P *˂ 0.05). Secondly, to eliminate multicollinearity and to reduce the number of IRGs to 20, LASSO Cox regression analysis was applied. (Fig. 3a–c, Additional file 1: Table S2, *P *˂ 0.05). Figure 3d and Fig. 3e depicted the network of interactions and the correlation between these IRGs. Thirdly, subsequent multivariable Cox regression analysis was used to construct a prognostic signature based on 7 IRGs (the C–C motif chemokine ligand 5 (*CCL5*), defensin alpha 4 (*DEFA4*), nuclear transcription factor Y subunit gamma (*NFYC*), estrogen receptor 1 (*ESR1*), tumor necrosis factor receptor superfamily member 8 (*TNFRSF8*), chemokine (C-X3-C motif) receptor 1 (*CX3CR1*), and serine protease inhibitor A3 (*SERPINA3*)) (Fig. 3f, Additional file 1: Table S3, *P* ˂ 0.05). As described earlier, the least AIC score aided in identifying of the IRGs signature (Table 2). The risk score was calculated as follows: − 0.465 × *CCL5* + 0.215 × *DEFA4 *− 1 .487 × *NFYC* + 1.055 × *ESR1 − *0.737 × *TNFRSF8 − *0.228 × *CX3CR1* + 1.003 × *SERPINA3* (AIC = 522.36, Concordance index = 0.78). Patients were classified into a low-risk group (*n* = 131) and a high-risk group (*n* = 108) based on the median cut-off value of their risk score (Fig. 4a). Additionally, there was no significant difference in clinical data between the high-risk and low-risk groups in the training cohort (Table 3). Fourthly, The Kaplan–Meier curve indicated that the low-risk group had a considerably increased survival than the high-risk group (Fig. 4c). Finally, the ROC curves shown that the area under the curve (AUC) was 0.876 [95% confidence interval (CI) 0.793–0.946] (Fig. 4e). The results presented the sensitivity was 0.893, the specificity was 0.874, the precision was 0.685, the precision was 0.685, the negative predictive value was 0.964, and the Matthews correlation coefficient (MCC) value was 0.706.

**Table 2:** Construction of prognostic signatures in patients with sepsis

Model	Prognostic signature combination	AIC
1	*JUN* + *CXCR3* + *CD3E* + *EDNRB* + *ADCYAP1R1* + *THBS1* + *HBEGF* + *TMSB10* + *KCNH2* + *PIK3CD* + *VEGFA* + *TRIM27* + *PTX3*	531.94
2	*JUN* + *CXCR3* + *CD3E* + *EDNRB* + *ADCYAP1R1* + *THBS1* + *HBEGF* + *TMSB10* + *KCNH2* + *PIK3CD* + *VEGFA* + *TRIM27* + *PTX3* + *CCL5*	530.70
3	*JUN* + *CXCR3* + *CD3E* + *EDNRB* + *ADCYAP1R1* + *THBS1* + *HBEGF* + *TMSB10* + *KCNH2* + *PIK3CD* + *VEGFA* + *TRIM27* + *PTX3* + *CCL5* + *DEFA4*	528.07
4	*JUN* + *CXCR3* + *CD3E* + *EDNRB* + *ADCYAP1R1* + *THBS1* + *HBEGF* + *TMSB10* + *KCNH2* + *PIK3CD* + *VEGFA* + *TRIM27* + *PTX3* + *CCL5* + *DEFA4* + *NFYC*	526.33
5	*JUN* + *CXCR3* + *CD3E* + *EDNRB* + *ADCYAP1R1* + *THBS1* + *HBEGF* + *TMSB10* + *KCNH2* + *PIK3CD* + *VEGFA* + *TRIM27* + *PTX3* + *CCL5* + *DEFA4* + *NFYC* + *ESR1*	526.00
6	*JUN* + *CXCR3* + *CD3E* + *EDNRB* + *ADCYAP1R1* + *THBS1* + *HBEGF* + *TMSB10* + *KCNH2* + *PIK3CD* + *VEGFA* + *TRIM27* + *PTX3* + *CCL5* + *DEFA4* + *NFYC* + *ESR1* + *TNFRSF8*	524.77
7	*JUN* + *CXCR3* + *CD3E* + *EDNRB* + *ADCYAP1R1* + *THBS1* + *HBEGF* + *TMSB10* + *KCNH2* + *PIK3CD* + *VEGFA* + *TRIM27* + *PTX3* + *CCL5* + *DEFA4* + *NFYC* + *ESR1* + *TNFRSF8* + *CX3CR1*	524.38
8	*JUN* + *CXCR3* + *CD3E* + *EDNRB* + *ADCYAP1R1* + *THBS1* + *HBEGF* + *TMSB10* + *KCNH2* + *PIK3CD* + *VEGFA* + *TRIM27* + *PTX3* + *CCL5* + *DEFA4* + *NFYC* + *ESR1* + *TNFRSF8* + *CX3CR1* + *SERPINA3*	524.34
9	*JUN* + *CXCR3* + *CD3E* + *EDNRB* + *ADCYAP1R1* + *THBS1* + *HBEGF* + *TMSB10* + *KCNH2* + *PIK3CD* + *VEGFA* + *TRIM27* + *CCL5* + *DEFA4* + *NFYC* + *ESR1* + *TNFRSF8* + *CX3CR1* + *SERPINA3*	524.32
10	*JUN* + *CXCR3* + *CD3E* + *EDNRB* + *ADCYAP1R1* + *THBS1* + *HBEGF* + *TMSB10* + *KCNH2* + *PIK3CD* + *VEGFA* + *CCL5* + *DEFA4* + *NFYC* + *ESR1* + *TNFRSF8* + *CX3CR1* + *SERPINA3*	524.09
11	*JUN* + *CXCR3* + *CD3E* + *EDNRB* + *ADCYAP1R1* + *THBS1* + *HBEGF* + *TMSB10* + *KCNH2* + *PIK3CD* + *CCL5* + *DEFA4* + *NFYC* + *ESR1* + *TNFRSF8* + *CX3CR1* + *SERPINA3*	524.08
12	*JUN* + *CXCR3* + *CD3E* + *EDNRB* + *ADCYAP1R1* + *THBS1* + *HBEGF* + *TMSB10* + *KCNH2* + *CCL5* + *DEFA4* + *NFYC* + *ESR1* + *TNFRSF8* + *CX3CR1* + *SERPINA3*	524.00
13	*JUN* + *CXCR3* + *CD3E* + *EDNRB* + *ADCYAP1R1* + *THBS1* + *HBEGF* + *TMSB10* + *CCL5* + *DEFA4* + *NFYC* + *ESR1* + *TNFRSF8* + *CX3CR1* + *SERPINA3*	523.98
14	*JUN* + *CXCR3* + *CD3E* + *EDNRB* + *ADCYAP1R1* + *THBS1* + *HBEGF* + *CCL5* + *DEFA4* + *NFYC* + *ESR1* + *TNFRSF8* + *CX3CR1* + *SERPINA3*	523.68
15	*JUN* + *CXCR3* + *CD3E* + *EDNRB* + *ADCYAP1R1* + *THBS1* + *CCL5* + *DEFA4* + *NFYC* + *ESR1* + *TNFRSF8* + *CX3CR1* + *SERPINA3*	523.46
16	*JUN* + *CXCR3* + *CD3E* + *EDNRB* + *ADCYAP1R1* + *CCL5* + *DEFA4* + *NFYC* + *ESR1* + *TNFRSF8* + *CX3CR1* + *SERPINA3*	523.39
17	*JUN* + *CXCR3* + *CD3E* + *EDNRB* + *CCL5* + *DEFA4* + *NFYC* + *ESR1* + *TNFRSF8* + *CX3CR1* + *SERPINA3*	523.09
18	*JUN* + *CXCR3* + *CD3E* + *CCL5* + *DEFA4* + *NFYC* + *ESR1* + *TNFRSF8* + *CX3CR1* + *SERPINA3*	523.66
19	*JUN* + *CXCR3* + *CCL5* + *DEFA4* + *NFYC* + *ESR1* + *TNFRSF8* + *CX3CR1* + *SERPINA3*	522.53
20	*JUN* + *CCL5* + *DEFA4* + *NFYC* + *ESR1* + *TNFRSF8* + *CX3CR1* + *SERPINA3*	522.40
21	*CCL5* + *DEFA4* + *NFYC* + *ESR1* + *TNFRSF8* + *CX3CR1* + *SERPINA3*	522.36

*AIC* Akaike information criterion

**Table 3:** Association between signature and clinical characters in the training cohort

	High-risk group (*n* = 108)		Low-risk group (*n* = 131)		*P*
Variables	No	%	No	%	
*Age (y)*					
≤ 65	58	53.70	80	61.09	0.293
> 65	50	46.30	51	38.91	
*Gender*					
Female	42	38.89	58	44.27	0.431
Male	66	61.11	73	55.73	
*Pneumonia*					
Community acquired pneumonia	21	19.44	37	28.24	0.076
Hospital acquired pneumonia	16	14.81	24	18.32	
Unknown	71	65.75	64	48.85	
*Thrombocytopenia*					
Yes	20	18.52	25	19.08	1.000
No	88	81.48	106	80.92	
*ICU-acquired infection*					
Yes	13	12.04	10	7.63	0.277
No	95	87.96	121	92.37	
*Diabetes*					
Yes	21	19.44	21	16.03	0.500
No	87	80.56	110	83.97	
*Abdominal sepsis*					
Yes	8	7.41	19	14.50	0.102
No	100	92.59	112	85.50	

*ICU* Intensive care unit

### Validation of the seven immune-related genes signature in the validation cohort

Similarly, patients were classified into a low-risk group (*n* = 119) and a high-risk group (*n* = 120) in the validation cohort (Fig. 4b). There was no significant difference in clinical data between the two risk groups in sepsis patients (Table 4). The Kaplan–Meier curve indicated that the low-risk group had a considerably increased survival than the high-risk group (Fig. 4d). The ROC curves indicate that the AUC was 0.855 (95% CI 0.781–0.932) (Fig. 4f). The results presented the sensitivity was 0.913, the specificity was 0.851, the precision was 0.663, the negative predictive value was 0.969, and the MCC value was 0.694 in the validation cohort. The expression levels of these 7 IRGs in the different outcomes groups were statistically significant (*P* < 0.05, Additional file 3: Fig. S2a–g). The verification of the external data set shows that the 28-day survival of the low-risk group is significantly higher than that of the high-risk group (Additional file 3: Fig. S2h) and the AUC of signature to predict 28-day survival in sepsis patients by risk score was 0.815 (*P* < 0.05, Additional file 3: Fig. S2i). The results presented the sensitivity was 0.889, the specificity was 0.789, the precision was 0.814, the negative predictive value was 0.872, and the MCC value was 0.682 in the external data set.

**Table 4:** Association between signature and clinical characters in the validation cohort

	High-risk (*n* = 120)		Low-risk (*n* = 119)			*P*
Variables	No	%	No		%	
*Age (y)*						
≤ 65	65	54.17	69		57.98	0.603
> 65	55	45.83	50		42.02	
*Gender*						
Female	51	42.50	55		46.22	0.604
Male	69	57.50	64		53.78	
*Pneumonia*						
Community acquired pneumonia	29	23.53		19	15.97	0.279
Hospital acquired pneumonia	17	14.29		20	16.81	
Unknown	74	62.18		80	67.22	
*Thrombocytopenia*						
Yes	20	16.67	17		14.29	0.721
No	100	83.33	102		85.71	
*ICU-acquired infection*						
Yes	11	9.17	12		10.08	0.830
No	109	90.83	107		89.92	
*Diabetes*						
Yes	20	16.67	27		22.69	0.259
No	100	83.33	92		77.31	
*Abdominal sepsis*						
Yes	13	10.83	9		7.56	0.381
No	107	89.17	112		92.46	

*ICU* Intensive care unit

### Independent prognostic value of seven immune-related genes signature

**Fig. 5:**
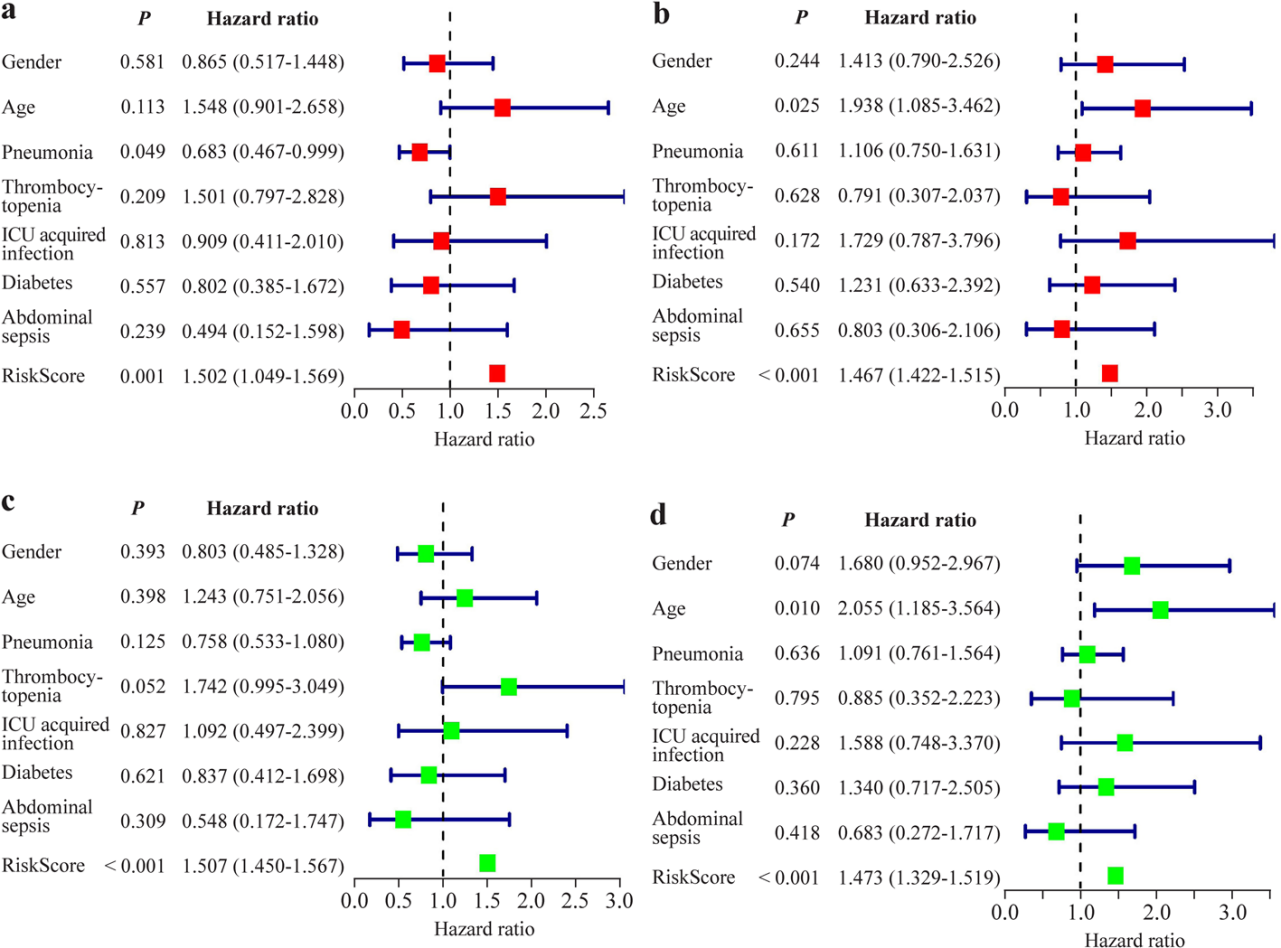
The immune-related genes signature has independent prognostic significance in both the training and validation cohorts. **a** Univariate Cox regression analysis of 28 days survival with risk scores and clinical information in the training cohort; **b** univariate Cox regression analysis of 28 days survival with risk scores and clinical information.in the validation cohort; **c** multivariable Cox regression analysis of 28 days survival with risk scores and clinical information in the training cohort; **d** multivariable Cox regression analysis of 28 days survival with risk scores and clinical information in the validation cohort. *ICU* Intensive care unit

Univariate Cox regression analysis revealed a correlation between the signatures of seven IRGs and the 28-day survival of sepsis patients (Fig. 5a and b). The 7 IRGs signature proved to be an independent prognostic factor in the multivariable Cox regression analysis (Fig. 5c, d).

### Enrichment analyses of the differentially expressed genes

**Fig. 6:**
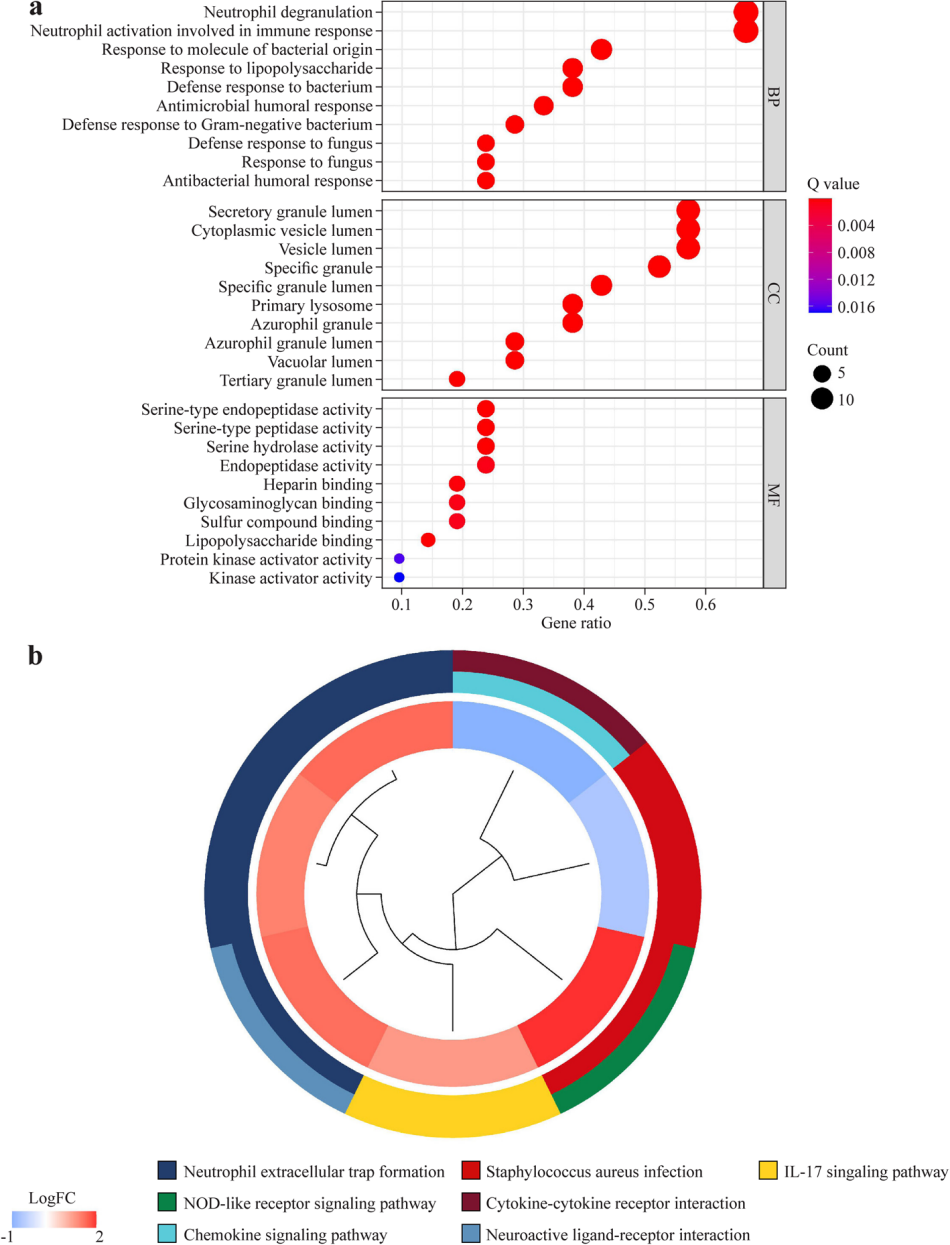
The results of GO and KEGG enrichment analysis of differentially expressed genes between different risk groups in sepsis patients. The most significant GO enrichment analysis; **b** the most significant KEGG pathways enrichment analysis [28]. *GO* gene ontology, *KEGG* Kyoto encyclopedia of genes and genomes, *IL* interleukin, *NOD* nucleotide-binding oligomerization domain, *FC* fold-change, *BP* biological process, *CC* cellular component, *MF* molecular function

The DEGs between low-risk group and high-risk group were shown in Additional file 1: Table S4. We conducted GO enrichment and KEGG pathway analyses on the DEGs to better understand their biological functions and pathways. The GO enrichment analysis revealed that DEGs were clearly enriched in immune-related functions, such as neutrophil degranulation and activation, which are implicated in the immunological response in sepsis patients. (Fig. 6a). In addition, the markedly enriched pathways for DEGs were neutrophil extracellular trap formation, staphylococcus aureus infection, interleukin (IL)-17 signaling pathway and nucleotide-binding oligomerization domain (NOD)-like receptor signaling pathway in sepsis patients (Fig. 6b). Inflammatory response (NES = 2.02, *P*. adjust < 0.05) was considerably enriched in the low-risk group patients of sepsis (Additional file 4: Fig. S3).

### The correlation between the risk score and immune status

**Fig. 7:**
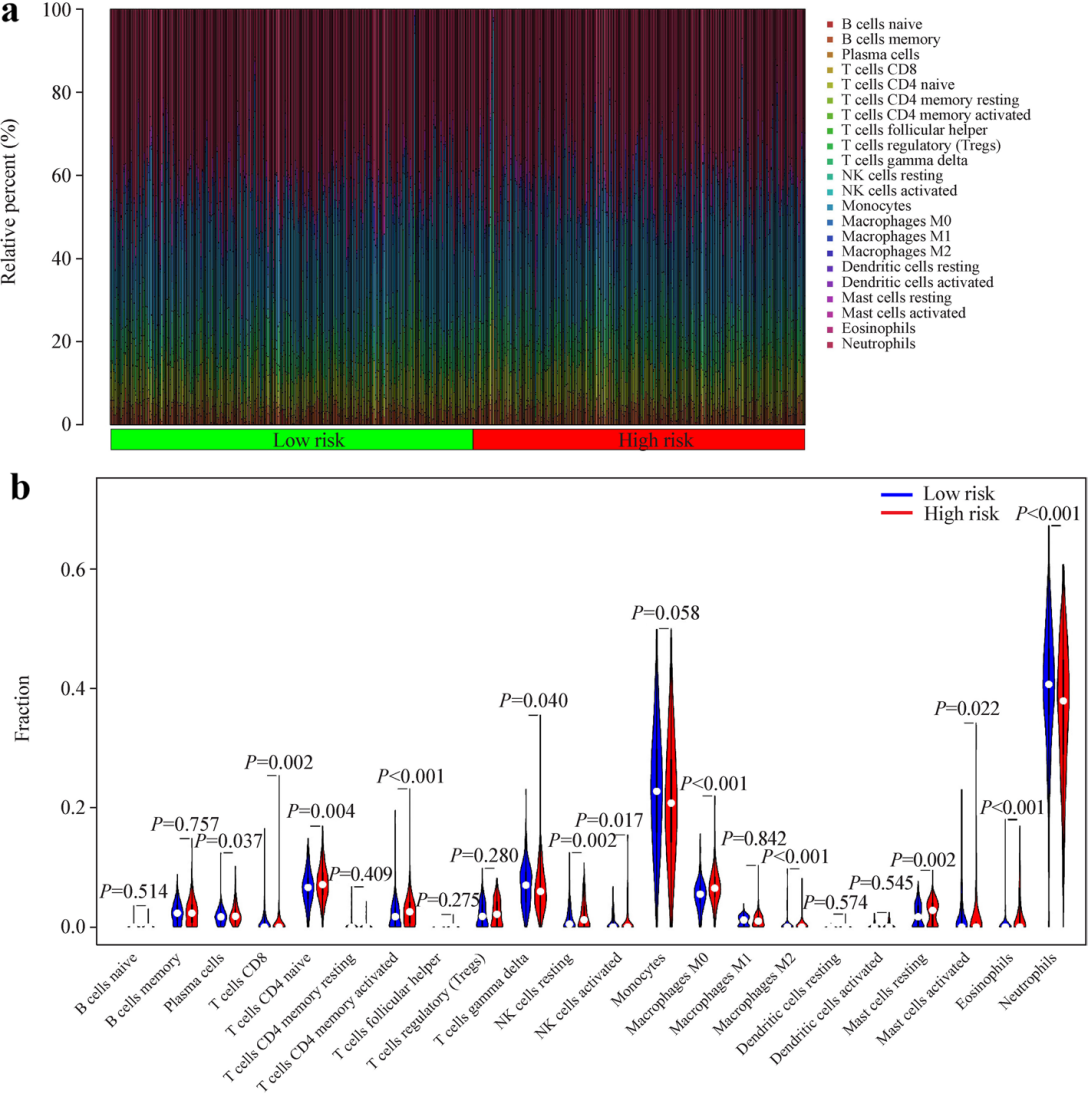
The immune cell infiltration landscape based on CIBERSORT algorithm between different risk groups in sepsis patients. Barplot (**a**) of the immune infiltrating cell proportions. Violin plot (**b**) demonstrated the differences in the proportions of immune infiltrating cell across different risk groups. *NK* natural killer

**Fig. 8:**
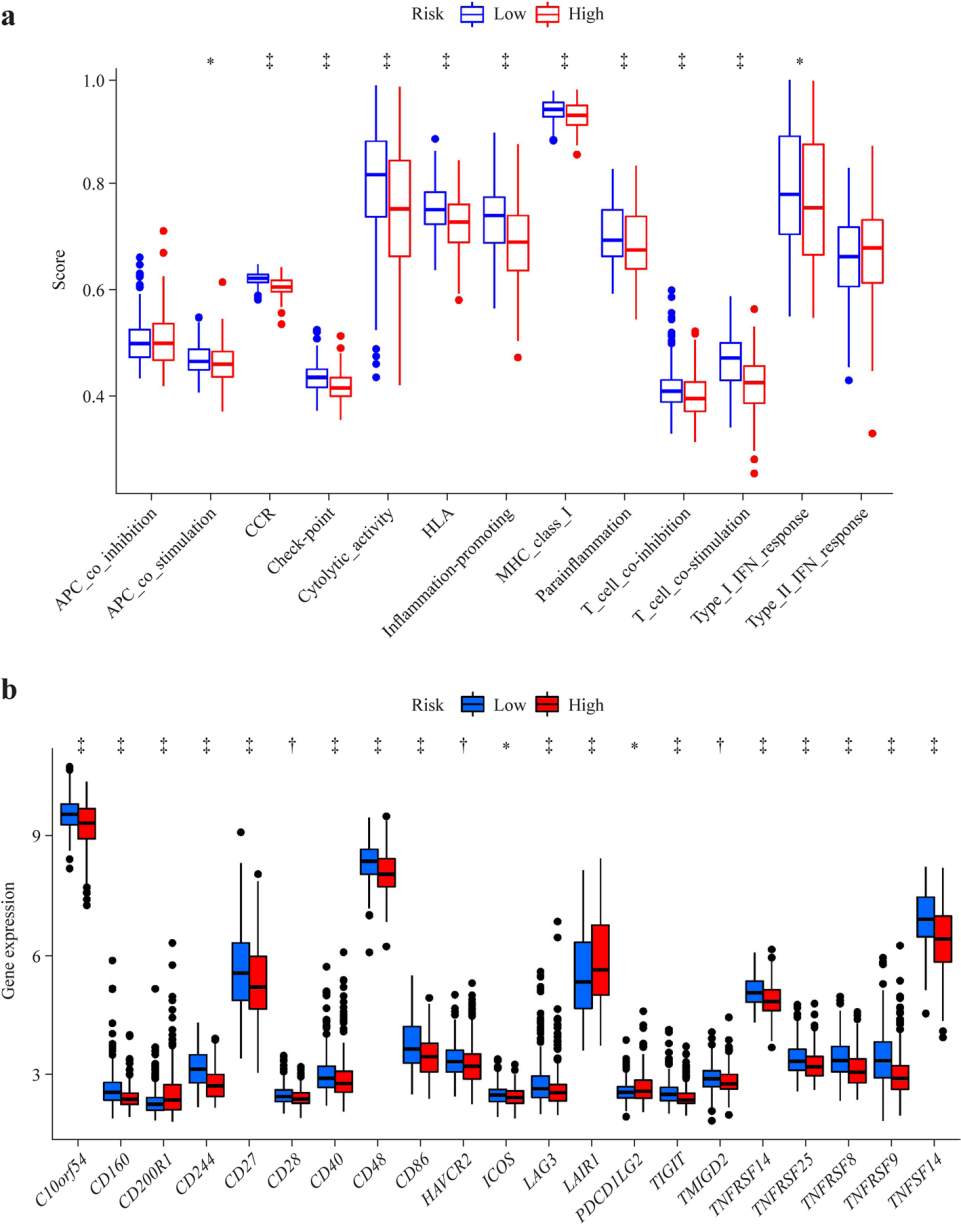
The single-sample gene set enrichment analysis scores of sepsis patients in different risk groups are compared. **a** The scores of immune functions; **b** the expression levels of immune checkpoints. *APC* allophycocyanin, *CCR* CC chemokine receptor, *HLA* human leukocyte antigen, *MHC* major histocompatibility complex, *IFN* interferon. ^*^P < 0.05, ^**^P < 0.01, ^***^P < 0.001

We explored the immune cell infiltration landscape while using results of the CIBERSORT algorithm. Patients with sepsis in the high-risk group had higher ratios of neutrophils, monocyte, and T cells CD8 than those in the low-risk group (*P* < 0.05, Fig. 7a, b). Patients with sepsis in the high-risk group had lower ratios of plasma cell, T cells CD4 naive, T cells CD4 memory activated, natural killer (NK) cells resting, NK cells activated, macrophages M0, macrophages M2, eosinophils, monocytes mast cells activated and mast cells resting than those in the low-risk group (*P* < 0.05, Fig. 7a, b). Next, the enrichment scores of a variety of immune related functions or pathways were calculated by ssGSEA. The score of CC chemokine receptor, check point, inflammation promoting and T cell co-stimulation were lower in the high-risk group in sepsis patients (*P* < 0.05, Fig. 8a). Interestingly, the fraction of neutrophils was the largest statistically significant difference between the high-risk and low-risk groups, which was consistent with the findings in the GO and KEGG analysis. In addition, the sepsis patients in the high-risk group were characterized by upregulated expression of *CD200R1* and leukocyte-associated immunoglobulin-like receptor 1, whereas the sepsis patients in the low-risk group were characterized by high expression of *C10orf54*, *CD160*, *CD244*, *CD40*, *CD48*, *CD86*, *LAG3*, *TIGIT*, *TNFRSF14*, *TNFRSF25*, *TNFRSF8*, *TNFRSF9* and *TNFSF14* (*P* < 0.05, Fig. 8b).

## Discussion

Several traditional prognosis indicators are applied in clinical practice today, including the Sequential Organ Failure Assessment [31], quick Sequential Organ Failure Assessment [32], the Acute Physiology and Chronic Health Evaluation II [33], the Simplified Acute Physiology Score II [34], and C-reactive protein/albumin ratio [35]. Nevertheless, their performances are limited in specificity and sensitivity so that they have facilitated early diagnosis and prognosis prediction in patients with sepsis [36].

Molecular biomarkers are considered as non-invasive clinical methods that could objectively predict or evaluate status and progression of disease. Regulation of the immune response and function is known to play a critical role in the pathogenesis and progression of sepsis [37, 38]. Wong et al. [39, 40] found that some genes associated with innate immune response could be used to predict the prognosis of children with clinical sepsis and showed good clinical efficacy. However, these authors did not systematically integrate these genes into a signature, making it impossible to use them in clinical practice. A study [41] verified that sepsis risk scoring methods based on multi-gene biomarkers showed higher performance (sensitivity and specificity) in the ROC curves. Therefore, by using univariate Cox regression analysis, it was determined that 126 IRGs were substantially linked with sepsis patient survival. In the end, we managed to formulate a signature of 7 IRGs for the prognosis of sepsis patients: − 0.465 × *CCL5* + 0.215 × *DEFA4 − *1.487 × *NFYC* + 1.055 × *ESR1 − *0.737 × *TNFRSF8 − *0.228 × *CX3CR1* + 1.003 × *SERPINA3*.

Among these 7 IRGs, which could predict the prognosis of sepsis patients, *CCL5*, *DEFA4*, *ESR1* and *CX3CR1* were broadly researched in previous studies. *CCL5*, a member of CC motif chemokines ligand, is recognized as an effective biomarker for the diagnosis of sepsis [42]. However, no study has found a relationship between CCL5 and the prognosis of sepsis. DEFA4, a member of the α-defensins family, has been shown to induce IL-6 release in macrophages in a toll-like receptor 4-independent manner [43]. Zhang et al. [44] elaborated that the expression of *DEFA4* is strongly correlated with the severity of sepsis. Moreover, *DEFA4* also may serve as a biomarker for clinical diagnosis and as a target for treatment of severe influenza infection [45]. *ESR1* has been reported as a biomarker of septic syndrome in patients with coronavirus disease 2019 [46]. *CX3CR1* is a G-protein coupled receptor, which is expressed on various cells, such as T lymphocytes, monocytes, natural killer cells, neurons and microglial cells [47]. Interestingly, not only is lower *CX3CR1* expression associated with early and late mortality in critically sick patients, but also with septic shock progression [48, 49]. Although little is known about the roles of *NFYC*, *TNFRSF8* and *SERPINA3* in sepsis, *NFYC* is characterized as a new regulator of skeletal muscle immunometabolic signaling [50]. *TNFRSF8/CD30* has been shown to inhibit the proliferation of autoreactive effector immune cells, hence assisting the body in resisting autoimmunity [51]. *SERPINA3* is also an inflammatory cytokine gene, which could induce significant lung injury after influenza infection [52]. Underlying mechanisms of these three genes in sepsis still need further explorations.

In addition, we conducted GO and KEGG enrichment analysis based on the DEGs between different risk groups and discovered that immune-related biological processes and pathways, such as neutrophil degranulation, neutrophil activation involved immune response, neutrophil extracellular trap formation, staphylococcus aureus infection, IL-17 signaling pathway and NOD-like receptor signaling pathway, were unveiled. Furthermore, our immune cell infiltration landscape results indicated neutrophils had lower infiltration than those sepsis patients in the high-risk group. Not only are neutrophils the first line of defense, armed with the ability to recognize and respond to infection in the absence of normal receptor expression, but they are also activated in sepsis to produce reactive oxygen species, nitric oxide, cytokines, proteases, and kinins. Xini et al. [53] discovered that an early absolute CD64/CD15/CD45 neutrophils count lower than 2500/mm^3^ is independently associated with unfavorable outcome of sepsis. Another study found that reduced neutrophil CD16 expression predicted an increased risk of death in critically ill patients with sepsis [54]. As for immune functions, our ssGSEA results indicated that the high-risk groups had slighter inflammation-promoting in both the training and the validation cohort. Previous research [6] revealed pro-inflammatory processes aid in the clearance of pathogenic agents in the initial phase of sepsis.

Our study still included some limitations. First, we did not employ additional prospective real-world data to validate our prognostic signature's clinical efficacy. Second, owing to a paucity of relevant clinical data, the predictive model we created did not incorporate all relevant clinical data. Finally, it was determined that the relationships between the risk score and immunological status should be validated empirically.

In conclusion, our work established a unique prognostic signature of 7 IRGs. In both the training and validation cohorts, this signature was found to be independently linked with survival, providing insight into the prediction of sepsis prognosis. The differences in neutrophil infiltration were found to be correlated to the progression of sepsis. Thus, in the future, this marker may develop into a viable biomarker for predicting sepsis, allowing for more sensible ICU resource distribution.

## Supplementary Information

**Additional file 1: Table S1.** The IRGs from the ImmPort database. **Table S2.** The 126 immune-related genes that were found to be linked with survival in individuals with sepsis. **Table S3.** The expression of 20 immune-related genes associated with survival in sepsis patients after LASSO Cox regression analysis. **Table S4.** The 7 immune-related genes associated with survival in sepsis patients after multivariate Cox regression analysis. **Table S5.** The differentially expressed genes between different risk groups in sepsis patients

**Additional file 2: Fig. S1.** Data preprocessing analysis: **a** PCA results after batch correction of gene expression profile in GSE65682; **b** PCA results after batch correction of gene expression profile in E-MATB-4451. *PCA* principal components analysis

**Additional file 3: Fig. S2.** The 7 IRGs signature's prognostic performance in the external dataset. The expression levels of **a** *CCL5*, **b** *DEFA4*, **c** *NFY4*, **d** *ESR1*, **e** *TNFRSF8*, **f** *CX3CR1*, and **g** *SERPINA3* in the different outcomes groups. **h** Kaplan–Meier curves depicting the 28-day survival based on the risk score in sepsis patients. **i** The prognostic accuracy of the risk score is confirmed by the AUC of ROC. *AUC* area under curve, *ROC* receiver operating characteristic curve, *P < 0.05, **P < 0.01, ***P < 0.001

**Additional file 4. Fig. S3.** Enrichment plots from GSEA between different risk groups patients with sepsis samples. *GSEA* gene set enrichment analysis

## Data Availability

The datasets analyzed during the current study are available in the Gene Expression Omnibus database (https://www.ncbi.nlm.nih.gov/genome/), and the Immunology Database and Analysis Portal database (https://www.immport.org/home).
