# Supplementary figures and images for: A signature of immune-related genes correlating with clinical prognosis and immune microenvironment in sepsis
Source: BMC Bioinformatics. 2023 Jan 17;24:20. doi: 10.1186/s12859-023-05134-1 (PMC9843880; doi:10.1186/s12859-023-05134-1)

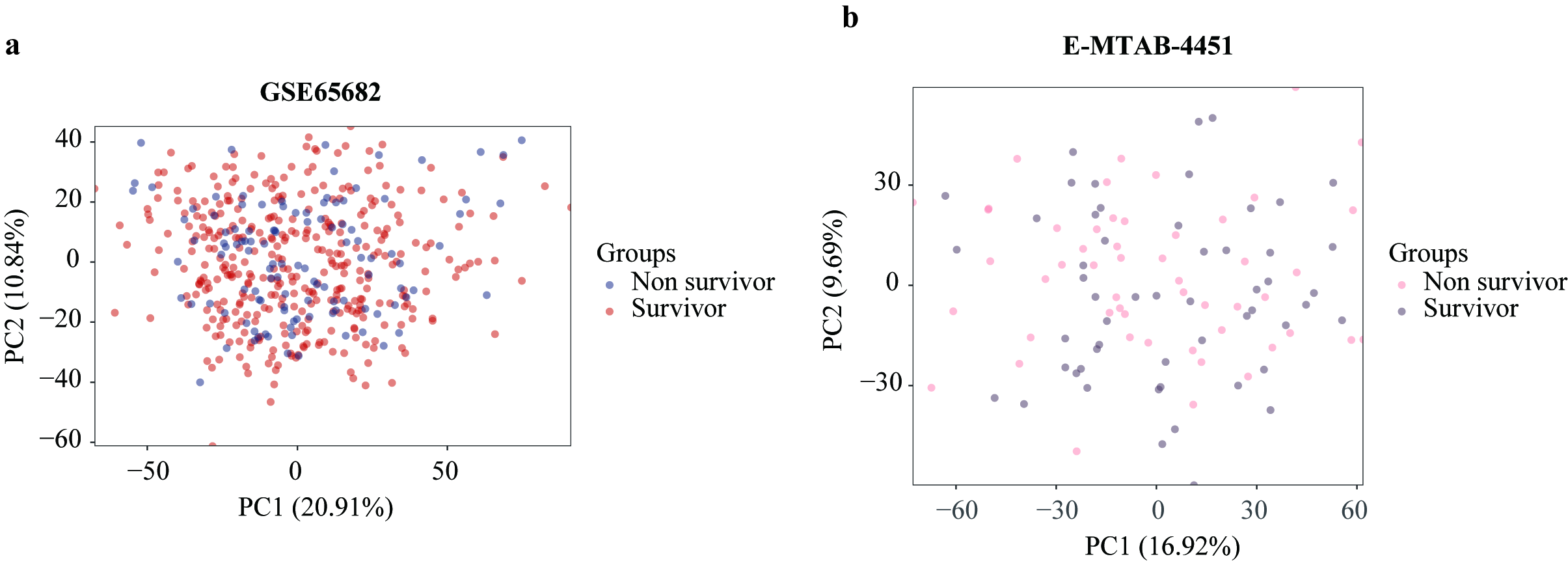

Supplement: Supplementary file 2 — Additional file 2: Fig. S1. Data preprocessing analysis: a PCA results after batch correction of gene expression profile in GSE65682; b PCA results after batch correction of gene expression profile in E-MATB-4451. PCA principal components analysis [file 12859_2023_5134_MOESM2_ESM.tif]

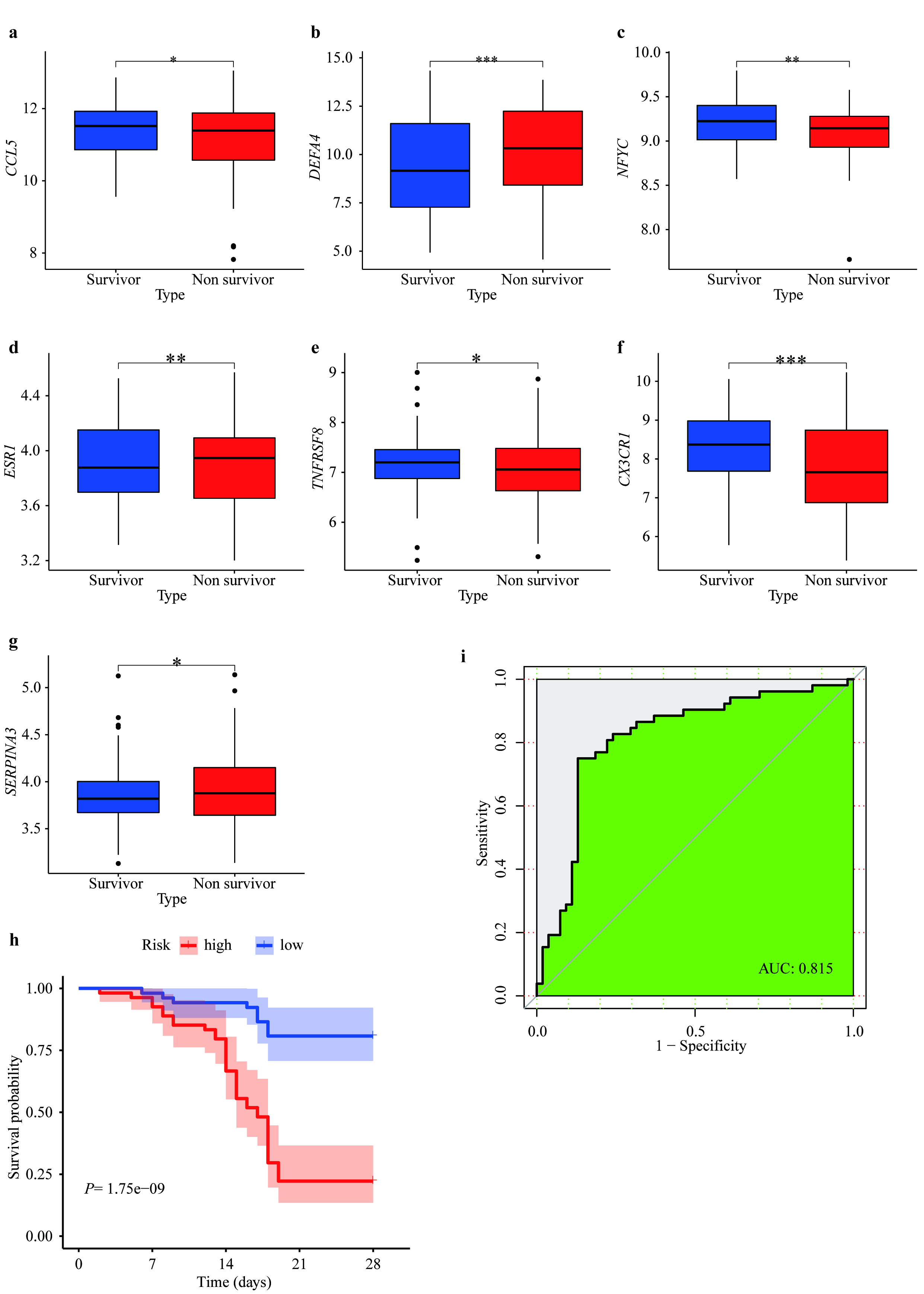

Supplement: Supplementary file 3 — Additional file 3: Fig. S2. The 7 IRGs signature's prognostic performance in the external dataset. The expression levels of a CCL5, b DEFA4, c NFY4, d ESR1, e TNFRSF8, f CX3CR1, and g SERPINA3 in the different outcomes groups. h Kaplan–Meier curves depicting the 28-day survival based on the risk score in sepsis patients. i The prognostic accuracy of the risk score is confirmed by the AUC of ROC. AUC area under curve, ROC receiver operating characteristic curve, *P < 0.05, **P < 0.01, ***P < 0.001 [file 12859_2023_5134_MOESM3_ESM.tif]

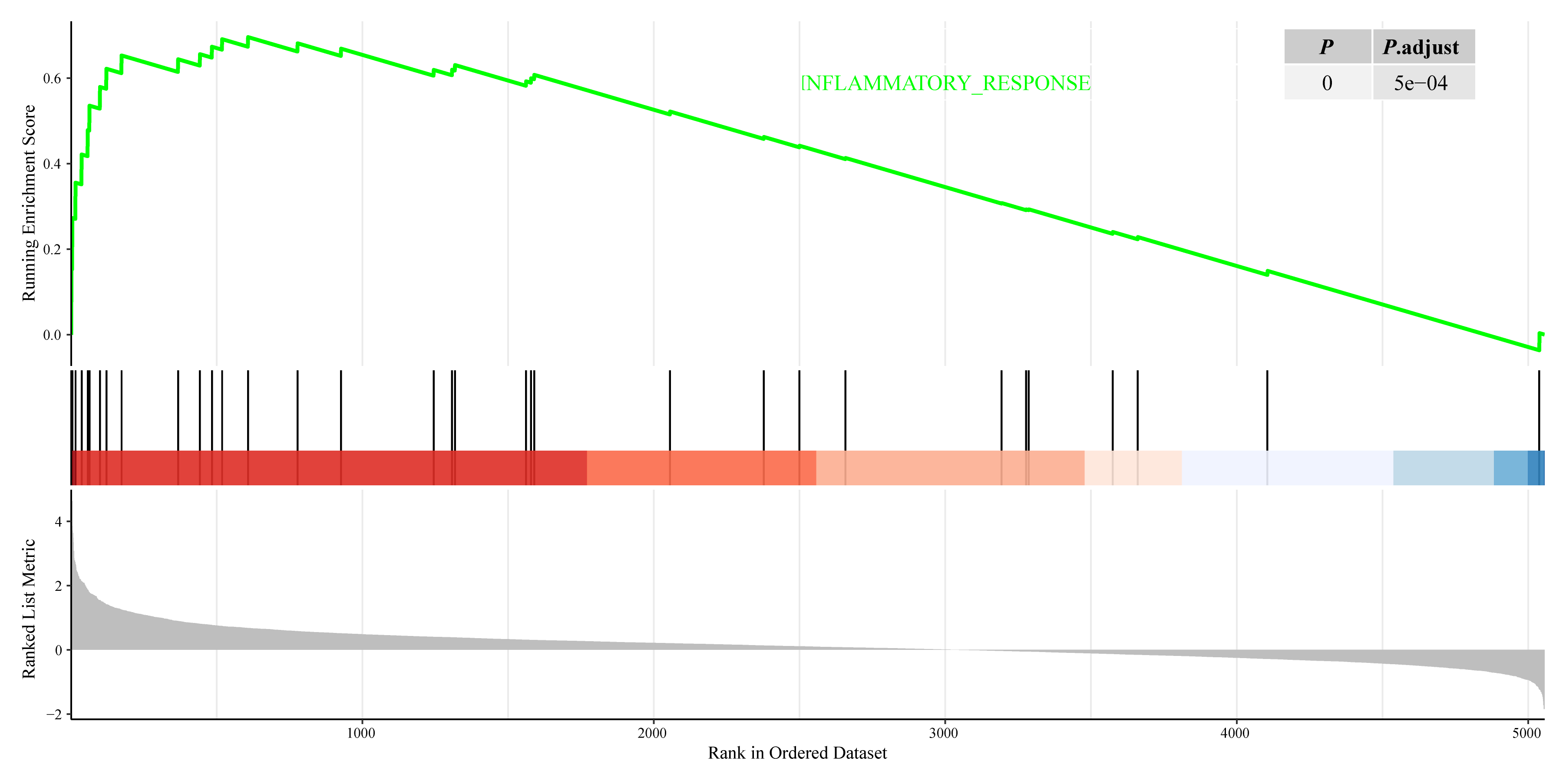

Supplement: Supplementary file 4 — Additional file 4. Fig. S3. Enrichment plots from GSEA between different risk groups patients with sepsis samples. GSEA gene set enrichment analysis [file 12859_2023_5134_MOESM4_ESM.tif]
